# A rapid screening method to select microdialysis carriers for hydrophobic compounds

**DOI:** 10.1371/journal.pone.0256920

**Published:** 2021-09-01

**Authors:** Sin-Jie Wang, Hsiao-Ting Lu, Yu-Chao Wang, Hsin-Ying Huang, Chung-Shi Yang

**Affiliations:** Institute of Biomedical Engineering and Nanomedicine, National Health Research Institutes, Zhunan, Miaoli, Taiwan; ISF College of Pharmacy, Moga, Punjab, INDIA

## Abstract

Microdialysis is a minimally invasive sampling technique which is widely applied in many fields including clinical studies. This technique usually has limitation on sampling hydrophobic compounds as aqueous solutions are commonly used as the perfusates. The relative recovery of hydrophobic compounds is often low and irreproducible because of the non-specific binding to microdialysis membranes or catheter tubing. Carriers such as cyclodextrins have been used to improve the recovery and consistency, however the identification of an optimal carrier can only be achieved after time-consuming and costly microdialysis experiments. We therefore developed a rapid, convenient, and low-cost method to identify the optimal carriers for sampling hydrophobic compounds with the use of centrifugal ultrafiltration. Doxorubicin was used as the model compound and its relative recoveries obtained from centrifugal ultrafiltration and from microdialysis were compared. The results show that the relative recoveries are highly correlated (correlation coefficient ≥ 0.9) between centrifugal ultrafiltration and microdialysis when different types or different concentrations of cyclodextrins were used as the carriers. In addition to doxorubicin, this method was further confirmed on three other drugs with different hydrophobicity. This method may facilitate and broaden the use of microdialysis perfusion on sampling or delivering hydrophobic substances in various applications.

## Introduction

Microdialysis is an *in vivo* sampling technique that allows collection and separation of analytes from complex biological environments [1]. Analytes of various molecular weights can be sampled with membranes of different molecular weight cut-off (MWCO) sizes [2]. Molecules of important functions like neurotransmitters, metabolites, or administrated drugs have been sampled and analyzed in different fields of biomedical researches, including clinical applications [3–6]. Although microdialysis sampling is a useful and valuable tool, it is usually applied on sampling of hydrophilic compounds with relatively low molecular weights [7].

Hydrophobic compounds are more difficult to be sampled by microdialysis perfusion [6], primarily because their RR_M_ (relative recoveries obtained from microdialysis) are low with large variation [8]. Two major approaches are usually used to overcome the limitations. One approach is to choose adequate materials of microdialysis probe and catheter tubing to lower the non-specific binding [9, 10]. The other approach is to add carriers or affinity agents such as surfactants [11], albumin [6], or cyclodextrins [7, 9] in perfusion solutions. Cyclodextrins are commonly used carriers because they are usually non-toxic [12], inexpensive, and have abundant thermodynamic binding data for numerous compounds [13].

There is a wide variety of cyclodextrins [14] that may be used as carriers for microdialysis applications. Selection of an optimal carrier [9, 15] can be achieved only after time-consuming and costly microdialysis trials. A rapid screening approach may facilitate this selection process, yet to the best of our knowledge there is no such approach to select carriers for hydrophobic analytes in the microdialysis experiments.

Therefore, we developed a rapid, convenient, and low-cost method to screen optimal carriers for sampling hydrophobic compounds with the use of centrifugal ultrafiltration which is a membrane-based separation or concentration technique [16, 17]. Doxorubicin was used as the model compound and its RR_U_ (relative recoveries obtained from ultrafiltration) and RR_M_ were compared when different types or concentrations of cyclodextrins were used as the carriers. We demonstrated that the RR_U_ are highly correlated with the RR_M_, thus the optimal type or concentration of carriers for microdialysis can be rapidly selected via a pre-run test by ultrafiltration, which may accelerate microdialysis investigations for sampling of hydrophobic molecules.

## Materials and methods

### Materials

α-CD (α-cyclodextrin), β-CD (β-cyclodextrin), γ-CD (γ-cyclodextrin), HP-α-CD (2-hydroxypropyl-α-cyclodextrin), HP-β-CD (2-hydroxypropyl-β-cyclodextrin), HP-γ-CD (2-hydroxypropyl-γ-cyclodextrin), doxorubicin, L-histidine, risperidone, sucrose, and temozolomide were obtained from Sigma (Saint Louis, Missouri, USA). Acetonitrile and methanol are products of J.T. Baker (Phillipsburg, New Jersey, USA) and Avantor Perf Mat—Macron Lab (Paris, Kentucky, USA) respectively. Abraxane (albumin-bound paclitaxel) was obtained from Celgene (Phoenix, Arizona, USA). Oasis HLB μElution SPE plates are products of Waters Co. (Milford, Massachusetts, USA).

### Microdialysis

A CMA400 syringe pump (CMA, Holliston, Massachusetts, USA) was used with a microsyringe MS-GAN250 (Exmire, Shizuoka, Japan) and a special 23G needle (15 mm, C-style, Exmire, Shizuoka, Japan). For sampling of doxorubicin, 10% sucrose solutions containing 10 mM L-histidine with or without cyclodextrins (saturated α-CD, saturated β-CD, saturated γ-CD, 60 mM HP-α-CD, 60 mM HP-β-CD, or 60 mM HP-γ-CD) were used as perfusion solutions. CMA20 microdialysis probes (100 kD MWCO) with 10 mm length were pre-equilibrated with the perfusion solutions. Doxorubicin (20–25 μg/mL) was dissolved in 10% sucrose containing 10 mM L-histidine and used as periprobe fluids. The perfusion flow rate was 0.5 μL/min. An initial dialysates from tubing dead volume were discarded, and the following dialysates were collected for 1 hour and assayed immediately. When different concentrations of γ-CD were tested, the concentration of doxorubicin in periprobe fluids was adjusted to 2.5 μg/mL.

The 10% sucrose solution containing 10 mM L-histidine was replaced to distilled water for sampling of risperidone (59–138 μg/mL) and temozolomide (1.1–1.3 mg/mL), and was replaced to saline for sampling of albumin-bound paclitaxel (5 mg/mL).

### Ultrafiltration

Ultrafiltration centrifuge tubes (Sartorius vivaspin6, 100 kD MWCO, Göttingen, Lower Saxony, Germany) were pre-equilibrated with the perfusion solutions mentioned in the microdialysis section. Drugs were dissolved in the periprobe solutions as mentioned in the microdialysis section and mixed equivalently with the perfusion solutions for 1 hour. The mixed solutions were assigned as starting solutions and placed into ultrafiltration centrifuge tubes followed by centrifugation at 2000xg for 4 minutes, and the ultrafiltrates were assayed immediately.

### Drug assay

The dialysates and ultrafiltrates were mixed with equal volume of acetonitrile or methanol for the following assays. Doxorubicin was quantified by fluorescence detection using an Infinite M200 microplate reader (TECAN, Männedorf, Zürich, Switzerland) with excitation wavelength of 480 nm and emission wavelength of 596 nm. Temozolomide and risperidone were assayed by absorption at wavelength of 330 nm and 278 nm respectively using the microplate reader.

Paclitaxel was quantified by UPLC (ultra-performance liquid chromatography) analysis. Samples were firstly mixed equivalently with acetonitrile and transferred to a HLB μSPE plate pre-conditioned with methanol and distilled water. The loaded plate was washed with 200 μL of distilled water twice and 200 μL of 5% methanol. Analytes were then eluted with methanol and evaporated by a Savant SpeedVac SPD121P vacuum concentrator (Thermo Scientific, Waltham, Massachusetts, USA). The residue was reconstituted in acetonitrile and mixed equivalently with distilled water, and 1 μL aliquot was injected to an Agilent 1290 Infinity UPLC system (Santa Clara, California, USA). The mobile phase was 50% acetonitrile, and the flow rate was 0.3 mL/min. Analytes were separated by an Agilent zorbax eclipse plus C18 column (2.1 mm x 50 mm, 1.8 μm id, Santa Clara, California, USA) and detected by absorption at wavelength of 230 nm (retention time: 2.07 min) as shown in S1 Fig. All drug standard solutions were prepared in corresponding solutions containing cyclodextrins or not.

### Calculation and statistics

Each experiment was executed at least in triplicate. Results were analyzed by OriginPro 2021 (Northampton, Massachusetts, USA) and expressed as mean ± standard deviation. The calculation of RR_M_ and the RR_U_ were adopted as previously described [9, 16] and defined as the following equations,

$${\mathrm{RR}}_{M}=\left(\frac{\mathrm{Compound}\;\mathrm{concentration}\;\mathrm{in}\;\mathrm{dialysate}}{\mathrm{Compound}\;\mathrm{concentration}\;\mathrm{in}\;\mathrm{initial}\;\mathrm{periprobe}\;\mathrm{fluid}}\right)\times 100\%$$

$${\mathrm{RR}}_{U}=\left(\frac{\mathrm{Compound}\;\mathrm{concentration}\;\mathrm{in}\;\mathrm{ultrafiltrate}}{\mathrm{Compound}\;\mathrm{concentration}\;\mathrm{in}\;\mathrm{starting}\;\mathrm{solution}}\right)\times 100\%$$

The Pearson’s correlation coefficient was used to establish correlations. Tests were performed by using OriginPro 2021.

### Results and discussion

To examine the effects of the carriers on sampling hydrophobic compounds, we firstly used carrier free solution to sample doxorubicin in periprobe solutions. The RR_M_ of doxorubicin range from 4.5% to 22.1% as shown in Table 1 and Table A in S1 Table. The mean RR_M_ is low (11.2 ± 6.2%) with large variation (RSD, relative standard deviation: 55.8%) which may be resulted from irreproducible non-specific binding to microdialysis probe [8]. We then added six types of cyclodextrins as carriers in the perfusion solutions for the microdialysis sampling. The RR_M_ of doxorubicin increase 2.0–4.4 folds when different cyclodextrins were added (Table 1). The RSD of RR_M_ also decrease from 55.8% to 4.4–18.1%. The increase of mean and decrease of RSD of the RR_M_ indicate that cyclodextrins can improve the microdialysis sampling on doxorubicin. Among these cyclodextrins, γ-CD was selected for further experiments because of its highest mean and lowest RSD of RR_M_. This selection conventionally can only be achieved by time-consuming and costly microdialysis works. Therefore, it is worthy to develop a rapid screening method to increase the throughput and lower the cost.

**Table 1:** RR_M_ and RR_U_ of doxorubicin when using different types of cyclodextrin as carriers.

	RR_M_ (%)		RR_U_ (%)	
	Mean ± SD	RSD	Mean ± SD	RSD
**No carrier**	11.2 ± 6.2	55.8	16.8 ± 0.8	4.5
**α-CD**	29.2 ± 3.7	12.6	26.1 ± 5.4	20.7
**β-CD**	27.5 ± 4.2	15.2	21.0 ± 3.0	14.5
**γ-CD**	49.5 ± 2.2	4.4	41.5 ± 2.4	5.8
**HP-α-CD**	21.9 ± 2.8	12.7	23.4 ± 1.8	7.9
**HP-β-CD**	40.8 ± 7.4	18.1	33.2 ± 3.0	9.1
**HP-γ-CD**	26.6 ± 2.7	10.2	32.8 ± 0.8	2.4

**Fig 1:**
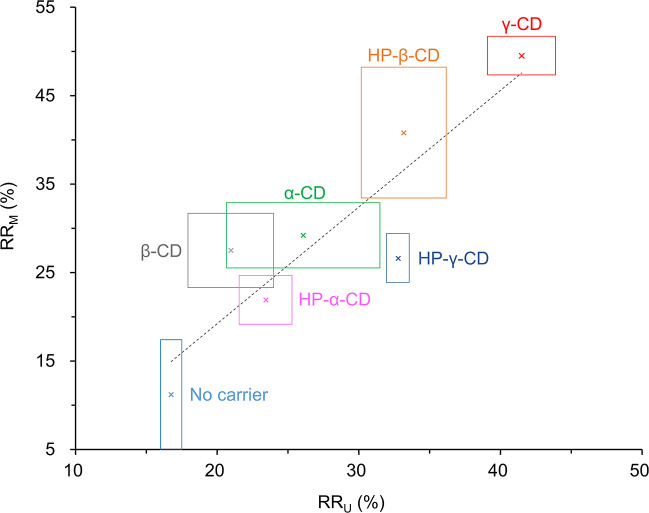
Correlation between the RR_U_ and the RR_M_ of doxorubicin when using different types of cyclodextrins as carriers. The cross marks represent the mean values, and the side lengths of the rectangles represent the SD. The Pearson’s correlation analysis shows a correlation coefficient of 0.90 (p = 0.006) which exhibits a high correlation between ultrafiltration and microdialysis.

We adopted centrifugal ultrafiltration which is a convenient and facilitated membrane-based separation technique to substitute microdialysis as a screening method. Doxorubicin was mixed with cyclodextrins and centrifugally ultrafiltrated to obtain their RR_U_. As shown in Table 1, the RR_U_ is the lowest (16.8 ± 0.8%) when no carrier is presented. When different cyclodextrins were added, the RR_U_ increase 1.3–2.5 folds. The sequence of RR_U_ levels is γ-CD > HP-β-CD > HP-γ-CD > α-CD > HP-α-CD > β-CD which is similar to the RR_M_. We compared their correlation in Fig 1 and found that the RR_U_ and the RR_M_ are highly correlated with a Pearson’s correlation coefficient of 0.90 (p = 0.006).

**Fig 2:**
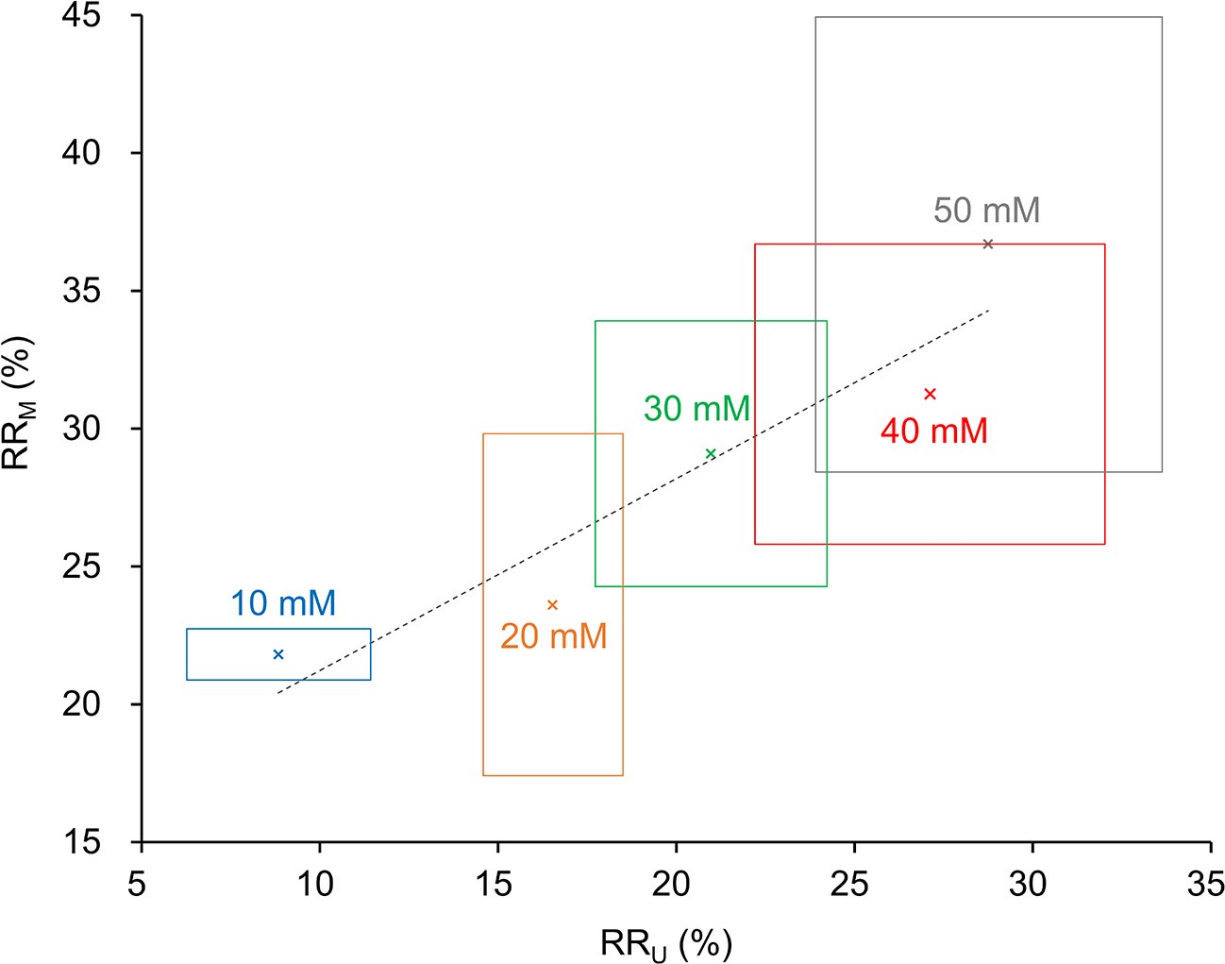
Correlation between the RR_U_ and the RR_M_ of doxorubicin when using different concentrations of γ-CD as carriers. The cross marks represent the mean values, and the side lengths of the rectangles represent the SD. The Pearson’s correlation analysis shows a correlation coefficient of 0.94 (p = 0.016) which exhibits a high correlation between ultrafiltration and microdialysis.

We then investigated the concentration effects of the selected γ-CD on sampling doxorubicin. Five different concentrations (10–50 mM) of γ-CD were used in perfusion fluids in microdialysis or added in starting solution in ultrafiltration. Both the RR_U_ and the RR_M_ of doxorubicin are enhanced with increasing γ-CD concentrations (Fig 2 and Table B in S1 Table). Fig 2 shows the RR_M_ are also highly and positively correlated with the RR_U_ with a correlation coefficient of 0.94 (p = 0.016). Herein we have proved the concept of using ultrafiltration as a screening method to predict the microdialysis outcome by demonstrating that the RR_M_ of doxorubicin are highly correlated with the RR_U_ (Figs 1 and 2). Both the optimal type and concentration of the carriers may be identified by this simple and rapid centrifugal ultrafiltration method.

**Fig 3:**
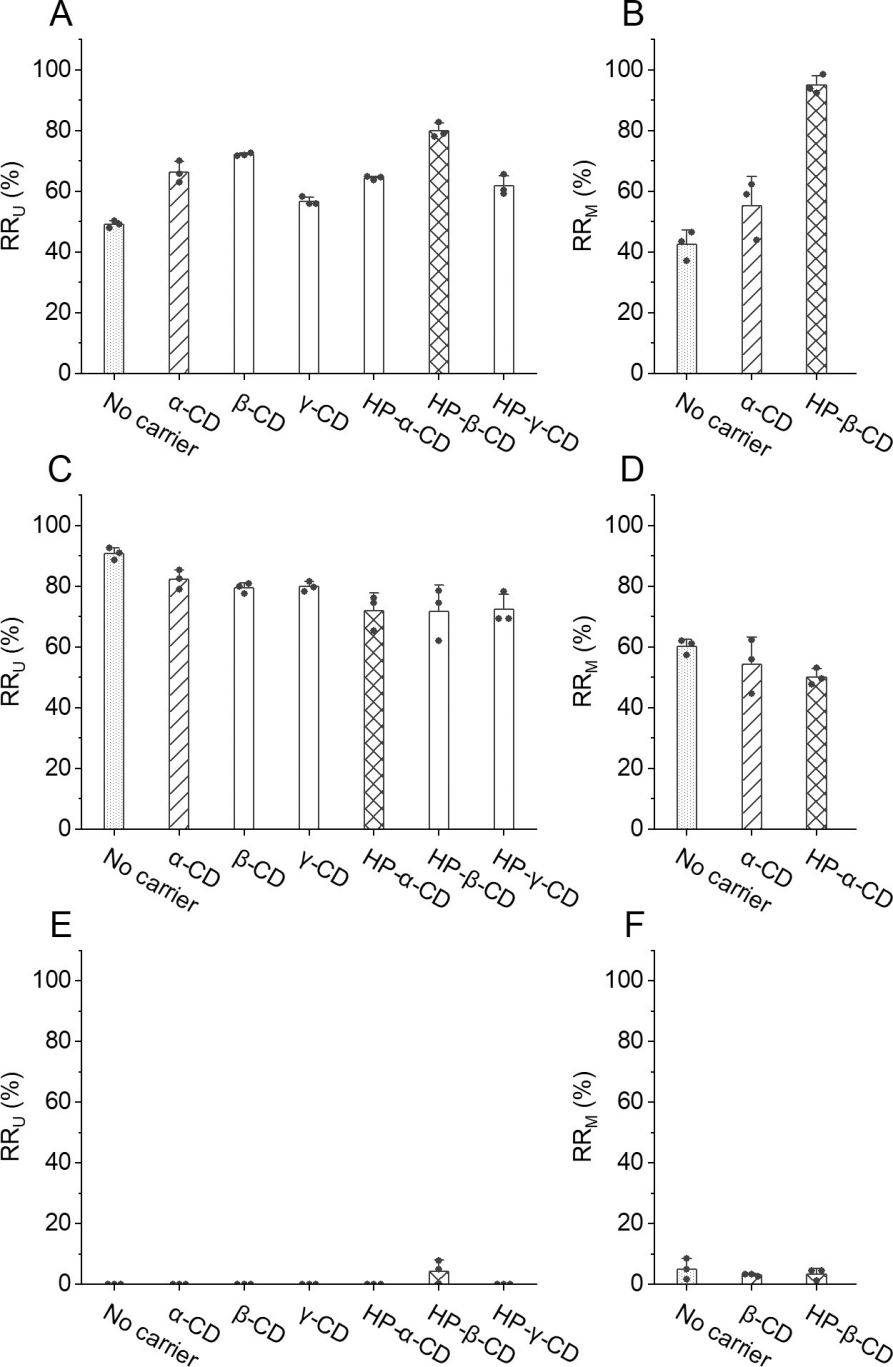
Carrier screening and method examination on three different drugs. The RR_U_ of (A) risperidone, (C) temozolomide, and (E) albumin-bound paclitaxel were tested and screened when different cyclodextrins were used in centrifugal ultrafiltration. Cyclodextrins with high, middle, and low RR_U_ in (A), (C), and (E) were selected to test their RR_M_ in (B), (D), and (F) to examine this screening method. The selected groups were respectively filled with dots, slashes, and cross lines. All experimental data are presented in solid circles.

We established this screening method based on the high correlation of the RR_U_ and the RR_M_ of doxorubicin whose logP is 1.27. We then explored the applicability of this method on another hydrophobic drug, risperidone, whose logP (3.27) is much higher than doxorubicin. When no carrier was added, the screening results show that the RR_U_ of risperidone is the lowest (49.1±1.2%), and the RR_U_ were enhanced when different cyclodextrins were added (Fig 3A and Table C in S1 Table). To examine the correspondence between microdialysis and ultrafiltration screening, HP-β-CD (the highest RR_U_), α-CD (middle RR_U_), and no carrier (the lowest RR_U_) were selected to test their RR_M_. The results show that the RR_M_ levels of risperidone is HP-β-CD > α-CD > no carrier (Fig 3B), which is the same as the ultrafiltration screening results.

In addition to risperidone, the same screening was performed on a less hydrophobic drug, temozolomide (water solubility = 5.09 mg/mL, logP = -2.8). When no carrier was added, the RR_U_ of temozolomide is high (90.7±2.0%) (Fig 3C) because temozolomide is relatively hydrophilic and thus can transport through the membrane well. When different cyclodextrins were added, the RR_U_ decreased a little to 71.7%-82.3% (Fig 3C and Table C in S1 Table). HP-α-CD (low RR_U_), α-CD (middle RR_U_), and no carrier (the highest RR_U_) were selected to test their RR_M_, and the results show that the RR_M_ of temozolomide is HP-α-CD < α-CD < no carrier (Fig 3D) which shows the same trend with the screening results. These results suggest that when sampling less hydrophobic compounds, carriers may not enhance the relative recoveries. Nevertheless, the screening method can still provide corresponding results for microdialysis.

We have tested three drugs with different logP (3.27 of risperidone, 1.27 of doxorubicin, and -2.8 of temozolomide), and the microdialysis results are all correspondent to the ultrafiltration screening results which exhibits centrifugal ultrafiltration is a good substitute for microdialysis. This screening method may be used to predict the microdialysis results and be applied to sampling of analytes with various hydrophobicity.

For hydrophobic drugs with very low stability and water solubility such as paclitaxel, it is difficult to solubilize the compound in water solution for the investigations. We chose a commercial formulated paclitaxel (albumin-bound paclitaxel) for the screening. The RR_U_ are all at low levels or below the detection limit with or without cyclodextrins (Fig 3E and Table C in S1 Table). Like the screening results, the RR_M_ are low (3.1%-5.1%) without significant differences when selected cyclodextrins were added (Fig 3F). Both the RR_U_ and the RR_M_ are very low probably due to the low mass transport of albumin (66 kD) against 100 kD MWCO membrane and low exchange efficiency of paclitaxel from albumin to cyclodextrins. Though the recoveries are limited, the ultrafiltration may still represent the condition of microdialysis. When the screening results exhibit low RR_U_, more other carriers shall be screened to identify the proper carriers.

Cyclodextrins are a family of cyclic oligosaccharides and composed of at least 150 species. Various types of cyclodextrin may be used as carrier candidates for hydrophobic drugs in microdialysis because they could form inclusion complexes with analytes and thus enhance the RR_M_ via facilitated mass transport [18]. While more than one species of cyclodextrins can be mixed to participate the host-guest chemistry [19], a fast and low-cost screening method shall be needed. As ultrafiltration centrifuge tubes are easy to use, low-cost consumables and multiple tubes can be centrifuged in parallel simultaneously, this screening method is expected to be useful and convenient. Besides cyclodextrins, there are many other types of carrier like surfactants or proteins that can be used in microdialysis experiments, and the extent of the applicability of this screening method needs to be further investigated.

## Conclusions

Centrifugal ultrafiltration can be used as a rapid screening method to identify the optimal types and concentrations of microdialysis carriers on sampling doxorubicin. The applicability of this screening method was further proved on sampling risperidone, temozolomide, and albumin-bound paclitaxel with various hydrophobicity. This method may facilitate the use of microdialysis perfusion on sampling or delivering hydrophobic compounds in various applications.

## Supporting information

S1 Fig The UPLC profile of paclitaxel with retention time of 2.07 min. (PDF)

S1 Table The original RR_M_ and RR_U_ of doxorubicin, risperidone, temozolomide, and albumin-bound paclitaxel when using different types of cyclodextrins as carriers. (PDF)
