# Supplementary figures and images for: A rapid screening method to select microdialysis carriers for hydrophobic compounds
Source: PLoS One. 2021 Sep 1;16(9):e0256920. doi: 10.1371/journal.pone.0256920 (PMC8409685; doi:10.1371/journal.pone.0256920)

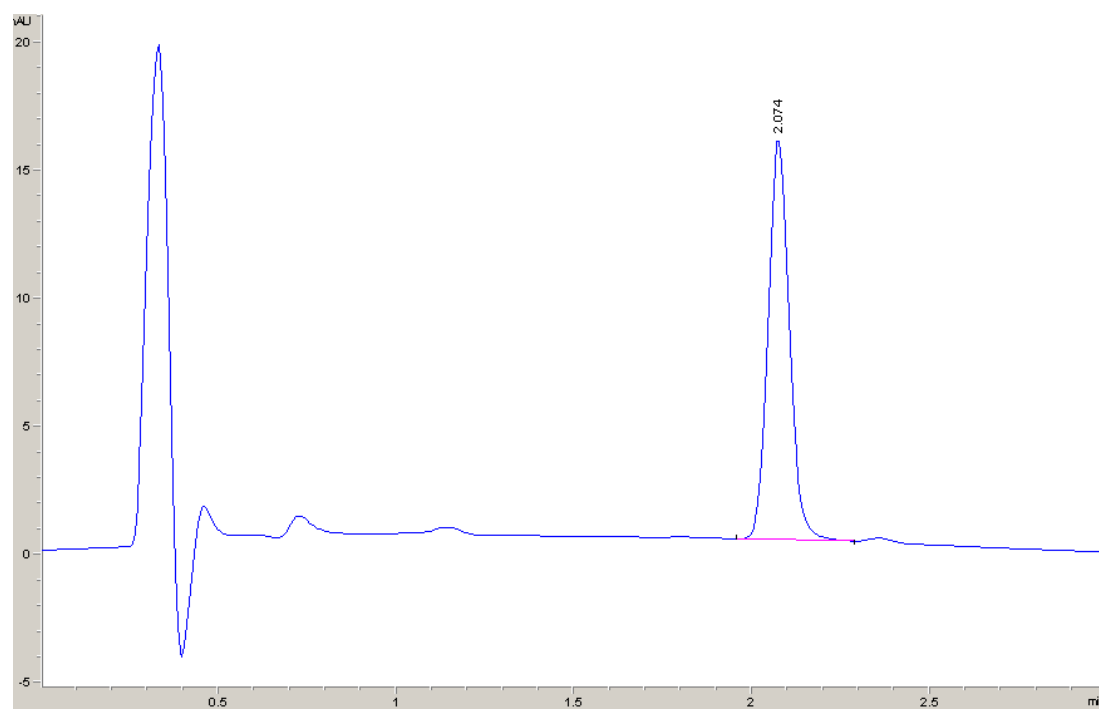

**S1 Fig. The UPLC profile of paclitaxel with retention time of 2.07 min.**

Supplement: S1 Fig — (PDF) [file pone.0256920.s001.pdf]
