# Supplementary material for: A rapid screening method to select microdialysis carriers for hydrophobic compounds
Source: PLoS One. 2021 Sep 1;16(9):e0256920. doi: 10.1371/journal.pone.0256920 (PMC8409685; doi:10.1371/journal.pone.0256920)
Supplement: S1 Table — (PDF) [file pone.0256920.s002.pdf]

**S1 Table. The original $RR_M$ and $RR_U$ of doxorubicin, risperidone, temozolomide, and albumin-bound paclitaxel when using different types or concentrations of cyclodextrins as carriers.**

**Table A. The original $RR_M$ and $RR_U$ of doxorubicin when using different types of cyclodextrins as carriers.**

| **Doxorubicin** | **$RR_M$ (%)** | | | | | | | | **$RR_U$ (%)** | | | |
|---|---|---|---|---|---|---|---|---|---|---|---|---|
| **Carriers** | Test 1 | Test 2 | Test 3 | Test 4 | Test 5 | Test 6 | Test 7 | Mean | Test 1 | Test 2 | Test 3 | Mean |
| **No carrier** | 9.3 | 16.7 | 22.1 | 10.9 | 4.5 | 5.7 | 8.9 | 11.2 | 16.2 | 16.5 | 17.6 | 16.8 |
| **α-CD** | 28.8 | 23.4 | 31.6 | 33.0 | 29.4 | - | - | 29.2 | 19.8 | 29.5 | 28.9 | 26.1 |
| **β-CD** | 24.5 | 25.6 | 32.2 | - | - | - | - | 27.5 | 18.6 | 24.4 | 19.9 | 21.0 |
| **γ-CD** | 49.5 | 47.9 | 52.5 | 48.0 | - | - | - | 49.5 | 41.0 | 39.4 | 44.1 | 41.5 |
| **HP-α-CD** | 24.7 | 19.1 | 21.9 | - | - | - | - | 21.9 | 21.9 | 22.9 | 25.5 | 23.4 |
| **HP-β-CD** | 40.0 | 47.1 | 49.5 | 32.9 | 34.4 | - | - | 40.8 | 36.6 | 31.8 | 31.1 | 33.2 |
| **HP-γ-CD** | 29.7 | 25.1 | 24.9 | - | - | - | - | 26.6 | 33.0 | 31.9 | 33.4 | 32.8 |

**Table B. The original $RR_U$ and $RR_M$ of doxorubicin when using different concentrations of γ-CD as carriers.**

| **γ-CD concentration** | **$RR_U$ (%)** | | | | **$RR_M$ (%)** | | | | | | |
|---|---|---|---|---|---|---|---|---|---|---|---|
| | Test 1 | Test 2 | Test 3 | Mean | Test 1 | Test 2 | Test 3 | Test 4 | Test 5 | Test 6 | Mean |
| **10 mM** | 7.6 | 11.8 | 7.1 | 8.8 | 21.8 | 22.7 | 20.9 | - | - | - | 21.8 |
| **20 mM** | 18.3 | 14.4 | 16.8 | 16.5 | 25.3 | 18.5 | 35.4 | 21.6 | 20.6 | 20.3 | 23.6 |
| **30 mM** | 17.2 | 22.3 | 23.3 | 21.0 | 34.0 | 28.9 | 24.3 | - | - | - | 29.1 |
| **40 mM** | 21.5 | 29.3 | 30.6 | 27.1 | 25.0 | 33.6 | 35.1 | - | - | - | 31.3 |
| **50 mM** | 28.8 | 23.9 | 33.6 | 28.7 | 34.8 | 48.5 | 30.7 | 41.3 | 28.2 | - | 36.7 |

**Table C. The original $RR_U$ and $RR_M$ of risperidone, temozolomide, and albumin-bound paclitaxel when using different types of cyclodextrins as carriers.**

| **Risperidone** | **$RR_U$ (%)** | | | | **$RR_M$ (%)** | | | |
|---|---|---|---|---|---|---|---|---|
| **Carriers** | Test 1 | Test 2 | Test 3 | Mean | Test 1 | Test 2 | Test 3 | Mean |
| **No carrier** | 50.3 | 49.2 | 47.8 | 49.1 | 37.2 | 46.6 | 43.4 | 44.4 |
| **α-CD** | 65.8 | 70.1 | 63.0 | 66.3 | 44.1 | 62.3 | 59.1 | 55.2 |
| **β-CD** | 72.1 | 71.7 | 72.6 | 72.1 | - | - | - | - |
| **γ-CD** | 55.9 | 58.3 | 55.9 | 56.7 | - | - | - | - |
| **HP-α-CD** | 63.8 | 64.8 | 64.6 | 64.4 | - | - | - | - |
| **HP-β-CD** | 79.0 | 82.9 | 78.0 | 80.0 | 92.5 | 93.8 | 98.5 | 94.9 |
| **HP-γ-CD** | 60.5 | 65.5 | 59.2 | 61.7 | - | - | - | - |
| **Temozolomide** | **$RR_U$ (%)** | | | | **$RR_M$ (%)** | | | |
| **Carriers** | Test 1 | Test 2 | Test 3 | Mean | Test 1 | Test 2 | Test 3 | Mean |
| **No carrier** | 88.7 | 90.9 | 92.6 | 90.7 | 61.1 | 62.0 | 57.5 | 60.2 |
| **α-CD** | 85.3 | 79.0 | 82.5 | 82.3 | 44.7 | 62.4 | 55.9 | 54.3 |
| **β-CD** | 80.9 | 77.6 | 80.0 | 79.5 | - | - | - | - |
| **γ-CD** | 79.7 | 81.7 | 78.2 | 79.9 | - | - | - | - |
| **HP-α-CD** | 65.3 | 76.3 | 74.4 | 72.0 | 49.5 | 47.6 | 53.0 | 50.1 |
| **HP-β-CD** | 74.5 | 78.5 | 62.0 | 71.7 | - | - | - | - |
| **HP-γ-CD** | 69.3 | 78.2 | 69.4 | 72.3 | - | - | - | - |
| **Albumin-bound paclitaxel** | **$RR_U$ (%)** | | | | **$RR_M$ (%)** | | | |
| **Carriers** | Test 1 | Test 2 | Test 3 | Mean | Test 1 | Test 2 | Test 3 | Mean |
| **No carrier** | ND | ND | ND | ND | 1.8 | 5.0 | 8.5 | 5.1 |
| **α-CD** | ND | ND | ND | ND | - | - | - | - |
| **β-CD** | ND | ND | ND | ND | 3.5 | 3.2 | 2.6 | 3.1 |
| **γ-CD** | ND | ND | ND | ND | - | - | - | - |
| **HP-α-CD** | ND | ND | ND | ND | - | - | - | - |
| **HP-β-CD** | 5.0 | 7.7 | 0.3 | 4.3 | 4.5 | 4.5 | 1.2 | 3.4 |
| **HP-γ-CD** | ND | ND | ND | ND | - | - | - | - |
